# Fish attraction to artificial reefs not always harmful: a simulation study

**DOI:** 10.1002/ece3.1730

**Published:** 2015-09-30

**Authors:** James A. Smith, Michael B. Lowry, Iain M. Suthers

**Affiliations:** ^1^ Evolution and Ecology Research Centre, and School of Biological, Earth and Environmental SciencesUniversity of New South WalesSydneyNSW2052Australia; ^2^ Sydney Institute of Marine ScienceChowder Bay RoadMosmanNSW2088Australia; ^3^ Port Stephens Fisheries InstituteLocked Bag 1Nelson BayNSW2315Australia

**Keywords:** Biomass redistribution, designed reefs, fish aggregation, fish production, fisheries enhancement, habitat quality

## Abstract

The debate on whether artificial reefs produce new fish or simply attract existing fish biomass continues due to the difficulty in distinguishing these processes, and there remains considerable doubt as to whether artificial reefs are a harmful form of habitat modification. The harm typically associated with attraction is that fish will be easier to harvest due to the existing biomass aggregating at a newly deployed reef. This outcome of fish attraction has not progressed past an anecdotal form, however, and is always perceived as a harmful process. We present a numerical model that simulates the effect that a redistributed fish biomass, due to an artificial reef, has on fishing catch per unit effort (CPUE). This model can be used to identify the scenarios (in terms of reef, fish, and harvest characteristics) that pose the most risk of exploitation due to fish attraction. The properties of this model were compared to the long‐standing predictions by Bohnsack (1989) on the factors that increase the risk or the harm of attraction. Simulations revealed that attraction is not always harmful because it does not always increase maximum fish density. Rather, attraction sometimes disperses existing fish biomass making them harder to catch. Some attraction can be ideal, with CPUE lowest when attraction leads to an equal distribution of biomass between natural and artificial reefs. Simulations also showed that the outcomes from attraction depend on the characteristics of the target fish species, such that transient or pelagic species are often at more risk of harmful attraction than resident species. Our findings generally agree with Bohnsack's predictions, although we recommend distinguishing “mobility” and “fidelity” when identifying species most at risk from attraction, as these traits had great influence on patterns of harvest of attracted fish biomass.

## Introduction

Artificial reefs can produce new fish biomass and attract existing fish biomass (Bohnsack 1989), but it is uncertain whether “production” or “attraction” is the dominant process (Bortone 1998, 2006). It is important to quantitate these processes, as attraction can cause aggregate existing fish biomass and make it easier to exploit (Grossman et al. 1997). The relative importance of production and attraction has fueled debate and research for decades (Bohnsack and Sutherland 1985; Lindberg 1997; Bortone 2006), and while there is a general understanding that it is a continuum and not a demarcation between attraction and production (Bohnsack 1989), there has been little quantitative information to inform the debate. Although progress is being made in identifying the processes and patterns of artificial reefs, using modern tools such as telemetry (Topping and Szedlmayer 2011; Piraino and Szedlmayer 2014), bioenergetics modeling (Shipley and Cowan 2011), and multispecies modeling (Campbell et al. 2011), the considerable uncertainty and risk of fish attraction have meant that the value of artificial reefs to fisheries enhancement remains in doubt (Powers et al. 2003; Cowan et al. 2011; Folpp et al. 2013).

For reefs open to fishing, it has been largely assumed that attraction of fish to an artificial reef is a harmful process, and species‐specific variation is usually ignored. Although numerous studies have attempted to quantitate or infer production (Powers et al. 2003; Brickhill et al. 2005; Leitão et al. 2007), few have modeled the exploitation risk caused by attraction (Brochier et al. 2015). So while attraction gained popularity due to the parsimony of the process for describing fish assemblages on artificial reefs, the impacts of attraction remained qualitative and uncertain. The great benefit of modeling attraction is that it can reveal consistent patterns with little knowledge of the system. And, unlike production, attraction is a straightforward concept (Carr and Hixon 1997) with fewer unknowns and assumptions.

Attraction is certainly an important process populating artificial reefs with fish. It is generally the case that artificial reefs develop adult fish assemblages quickly after deployment (Walsh 1985; Pickering and Whitmarsh 1997; Folpp et al. 2011), and this fits most definitions of “attraction.” Causes of attraction may be simple “thigmotaxis” (Brickhill et al. 2005) or “instinctive orientation” (Pickering and Whitmarsh 1997), but the “behavioral preferences” often listed as a cause of attraction (Bohnsack 1989) may not be distinct from fish production. If a newly deployed artificial reef offers some increased value to the attracted fish, such as improved refuge, then there may also be an increase in fish production through increased growth or survival. The risk of exploitation is equal to whether the increase in fish harvest (influenced by attraction) is more than that the increase in fish production (Lindberg 1997), but estimating this risk requires distinguishing redistribution with and without benefit to production. This is notoriously difficult, but an alternative is to model attraction and subsequent fishing catch. Until we are better able to model and predict production across a range of scenarios, we need to have a better understanding of the risks of attraction.

If it is assumed that attraction is the only process altering fish biomass at a reef, then total fish biomass in the surrounding area is the same before and after reef deployment (Brickhill et al. 2005). This same biomass can be distributed according to factors influencing attraction, namely distance to natural reef, reef size, and relative reef quality, and some characteristics of the associated fish species regarding their distribution and ability to detect and move to a new reef. This choice of factors allowed a quantitative evaluation of the qualitative predictions proposed in a seminal article by Bohnsack (1989) on the scenarios at greatest risk from attraction; that is, high reef availability, high fishing intensity, partial reef dependency, and pelagic‐like migratory behavior.

In this article, we present a numerical model that simulates how a redistributed fish biomass, due to an artificial reef, alters fishing catch per unit effort. It identifies the scenarios of reef, fish, and harvest characteristics for which the risk of exploitation due to fish attraction is highest. A goal is to further the “production versus attraction” debate, by showing that attraction is a complex process that is not always harmful and should be quantitatively evaluated in data‐poor systems when exploitation of fisheries on artificial reefs is a concern.

## Methods

This attraction model was designed to model the distribution of fish biomass around a natural reef and then to model the redistribution of this same biomass after the addition of an artificial reef. Given that attraction is the only process being modeled, the total fish biomass before and after reef deployment is equal – it is only redistributed. The metric used to evaluate the impact of attraction was catch per unit effort (CPUE). So, for a given level of effort, the catch before reef deployment was compared to the catch after. If CPUE increased after reef deployment, we considered this potentially harmful and evidence toward an artificial reef increasing the risk of exploitation of fish biomass. By varying the multiple factors that can influence the magnitude of fish attraction, it was possible to identify scenarios in which the risk of exploitation is greatest.

The depletion of natural reefs due to attraction is a key component of the “attraction issue,” and this is the focus of this study. We consider two types of reef‐associated fish, one that is reef obligate, such as mado *Atypichthys strigatus* (Scott et al. 2015), and one that exists in the pelagic environment but associates with reefs, such as yellowtail scad *Trachurus novaezelandiae* (Scott et al. 2015). The structure of the model is described below and summarized in Table 1. This is a spatially discrete model, implemented using 10 × 10 m cells and an arbitrary system size of 211 × 211 cells. The model was designed as a demonstration model (Evans et al. 2013) to reveal generalities and explore parameter importance rather than to model a specific system.

**Table 1:** Descriptions of the parameters varied during model simulations, and the range of values explored. Note that not all parameters were varied simultaneously, and scrutiny of multiple figures is necessary to evaluate simulation results

Parameter	Definition	Relevance	Values used in simulations
*D*_*h*_	Distance from reef at which fish abundance halves (fidelity)	Describes the strength of the association between fish and a reef; resident prey species will have a smaller *D*_*h*_ than large transient species	7 distances: 10, 15, 20, 25, 30, 35, 40 m
*Q*	Relative reef quality	This represents the attractiveness of the artificial reef; increasing this value increases the number of fish that will leave their natural reef. *Q *=* *5 means that an artificial reef will have 5 times the biomass density of an immediately adjacent natural reef	10 values: 1–10
*D*_50_	The distance at which half the fish biomass will be attracted to an artificial reef (mobility)	This parameter is needed to ensure attraction declines with distance between reefs; *D*_99_ was kept at a constant value of twice *D*_50_	2 values: 300 m for reef residents, 200 m for reef‐associated pelagics
*D*_*r*_	Distance between natural and artificial reefs	By varying the distance between the artificial and natural reefs, it is possible to explore the role of partial attraction on CPUE	12 distances, approx: 42, 85, 127, 170, 212, 255, 297, 339, 382, 424, 467, 509 m
*S*_*r*_	Size of natural reef	By keeping a constant 1 × 1 cell artificial reef and varying the natural reef size, we could explore the role of fish density on attraction and CPUE	1 × 1 cell, 2 × 2 cells, 3 × 3 cells
*p*	Number of cells being fished simultaneously	This parameter represents fishing effort; as *p* increases, cells with lower fish biomass are increasingly fished; as *p* gets very large *CPUE*_*a*_: *CPUE*_*b*_ approaches 1 (because most fish are being caught, wherever they are)	9 values: 2, 5, 10, 20, 30, 40, 50, 70, 100

### The distribution and attraction of fish biomass

The first step in the model was to distribute fish biomass around a natural reef. The decline of biomass with increasing distance from a reef was defined by an exponential function, based on research that shows this can approximate the change in abundance with distance (dos Santos et al. 2010):

(1) $$B_{i}=e^{-k_{D_{h}}D_{i}}$$

where *B*_*i*_ is relative biomass in cell *i*, $k_{D_{h}}$ is a shape parameter for distance *D*_*h*_, and *D*_*i*_ is the distance from cell *i* to a given reef. This equation was also used to define the distribution of fish biomass around the artificial reef. The shape parameter was calculated using the distance from reef (*D*_*h*_) at which biomass density halves and is given as follows:

(2) $$k_{D_{h}}=\frac{\ln2}{D_{h}}$$

The second step was to add an artificial reef and calculate the attracted fish biomass. The reef was deployed at a variable distance *D*_*r*_ from the natural reef, which was used to calculate *D*_*i*_. We defined attraction as a function of distance between the natural and artificial reefs (closer reefs attract more fish) and of relative reef quality (higher quality artificial reefs will attract more fish). A logistic function (Smith and Taylor 2014) was used to define the amount of fish biomass in a given cell that is attracted to the artificial reef:

(3) $$B_{r_{i}}=B_{i}-B_{i}\left(Q_{0}{\left(1+e^{\frac{\ln(99^{2})}{D_{99}}\left(D_{i}-D_{50}\right)}\right)}^{-1}\right)$$

where $B_{r_{i}}$ is the relative biomass remaining in cell *i* after attraction, *Q*_0_ is a reef quality parameter, *D*_99_ is the distance over which almost all (99%) attraction occurs, and *D*_50_ is the distance at which half the fish in a cell will be attracted a reef. *D*_99_ was fixed at twice the value of *D*_50_ to ensure a symmetric curve. The logistic was chosen given its flexibility of shape and the logic of the *D*_50_ parameter for decision‐based processes, but other functions could be used. Reef quality was simply used in this model to define a reef's attractiveness which, ecologically, means appealing to the “behavioral preferences” of a species (Bohnsack 1989) without increasing fish production. *Q*_0_ defines the starting point of the logistic function and represents the proportion of the fish biomass attracted away from a natural reef as *D*_*i*_ approaches zero; meaning that we can vary the proportion of fish that will leave the natural reef. This can be expressed in terms of a ratio (*Q*) that defines the relative biomass between artificial and natural reefs as *D*_*i*_ approaches zero:

(4) $$Q=\frac{Q_{0}}{1-Q_{0}}$$

(5) $$Q_{0}={\left(\frac{1}{Q}+1\right)}^{-1}$$

For example, when *Q *=* *2 (*Q*_0_ = 2/3), an artificial reef will have twice the biomass of an immediately adjacent natural reef after attraction occurs.

**Figure 1:**
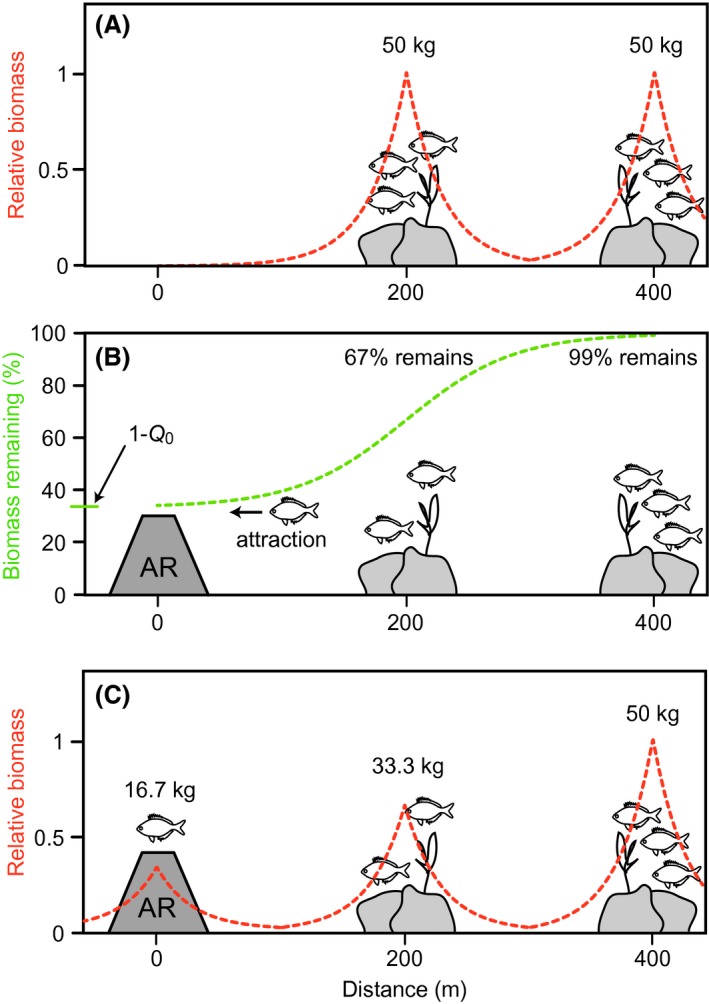
A hypothetical example illustrating the two mathematical functions that drive the distribution and attraction of fish biomass in this study. (A) two natural reefs before artificial reef deployment with equal biomasses of fish, distributed with distance from their natural reef according to an exponential function (equation (1), red line); (B) an AR is deployed and attraction occurs according to a logistic function (equation (3), green line); the closer an NR is to the AR, the more attraction occurs (i.e., the less biomass remains on the NR), to a maximum attraction of “*Q*_0_” when distance approaches zero (equation (5)); (C) the attracted fish biomass is then distributed around the artificial reef according to the exponential function (red line). In this example, 100 kg of fish is in the system and 15% is redistributed due to attraction to the artificial reef. The parameter values used were *Q *=* *2 (*Q*_0_ = 2/3), *D*_*h*_ = 20, *D*_50_ = 200, and *D*_99_ = 400.

The final step was to redistribute, around the artificial reef, the biomass that was attracted to the artificial reef. This was performed by summing, for all *n* cells in the modeled system, the biomass that was attracted to the artificial reef and redistributing it based on cell *i*'s relative biomass density (according to eq. (1)):

(6) $$B_{a_{i}}=B_{r_{i}}+\sum_{i=1}^{n}\left(B_{i}-B_{r_{i}}\right)\frac{B_{i}}{\sum_{i=1}^{n}B_{i}}$$

where *B*_*ai*_ is the biomass in cell *i* after attraction. For occasions where a specific cell had biomass associated with more than one reef cell (such as very close natural and artificial reefs, or natural reefs larger than 1 × 1 cell), the biomass $B_{a_{i}}$ was estimated as the maximum biomass for that cell (i.e., the response of fish biomass to reefs was not additive). Figure 1 illustrates a hypothetical example in which two natural reefs are depleted by attraction to an artificial reef, with equation (1) defining the distribution of fish biomass around each reef and equation (3) defining the biomass remaining after attraction.

### Measuring CPUE

CPUE was the metric used to examine the risk of exploitation of the fish biomass due to attraction to an artificial reef. CPUE was measured in this model as the sum of the fish biomass in a given number of cells (*p*) with the largest biomass. Thus, fishing effort is related to the size of *p*, as this represents how many cells are simultaneously being fished. Exploitation risk can be evaluated by comparing the CPUE before (*CPUE*_*b*_) and after (*CPUE*_*a*_) artificial reef deployment:

(7) $${\text{CPUE}}_{b}=\sum_{i=1}^{p}B_{i}$$

(8) $${\text{CPUE}}_{a}=\sum_{i=1}^{p}B_{a_{i}}$$

This formulation assumes that CPUE is linearly related to fish biomass density, that fishing effort per unit area is constant and effort is varied by changing the amount of area (i.e., cells) fished, and that fishers will always fish the cell(s) with the largest fish biomass. These assumptions were made to generalize the “worst case scenario.” However, CPUE is not always linearly related to biomass density (Beverton and Holt 1957; Campbell 2004), so an evaluation of a threshold CPUE model (maximum CPUE occurs before maximum fish density) was also explored. This type of model could arise given a fixed handling time (Hilborn and Walters 1987). The assumption of a fixed effort per unit area means that the amount of area being fished before and after reef deployment is the same, only redistributed. This was an appropriate level of detail for this model and targets the effect of reef size on CPUE given that larger reefs can be exposed to more simultaneous fishing effort. The assumption of fishers always fishing the highest biomass cells is a gravity model (Christensen and Walters 2004; Grüss et al. 2011) with no uncertainty – again to model the worst case scenario. The ratio *CPUE*_*a*_: *CPUE*_*b*_ was used to evaluate exploitation risk, and when this ratio >1, the attraction of fish to the artificial reef has increased CPUE for a given level of fishing effort (*p*).

### Modeling reef residents and reef‐associated pelagics

It is important to allow for species‐specific responses in attraction to an artificial reef, and the parameters *D*_*h*_, *D*_50_, and *D*_99_ can be varied to address this. Another important factor is the degree of residency. The model formulation presented above operates for resident reef species, but these are not the only fish group that associates with artificial reefs (Scott et al. 2015); in particular, transient or pelagic species can associate with reefs in great densities (Smith et al., unpublished data). These species can exist in the pelagic zone in the absence of a reef, but the model formulation above assumes that biomass asymptotes to zero away from any reef (eq. (1)). To use this model for reef‐associated pelagic species, which are not reef dependent, an additional step was required. This step was to create the modeled system without any reefs and assume a uniform biomass distribution across all *n* cells, but then add the natural reef and calculate attraction of the pelagic biomass to this natural reef (as was done above for the artificial reef, but assuming a constant quality *Q*_*nr*_ = 1). This was then followed by a second round of attraction due to the addition of the artificial reef.

### Simulations

**Figure 2:**
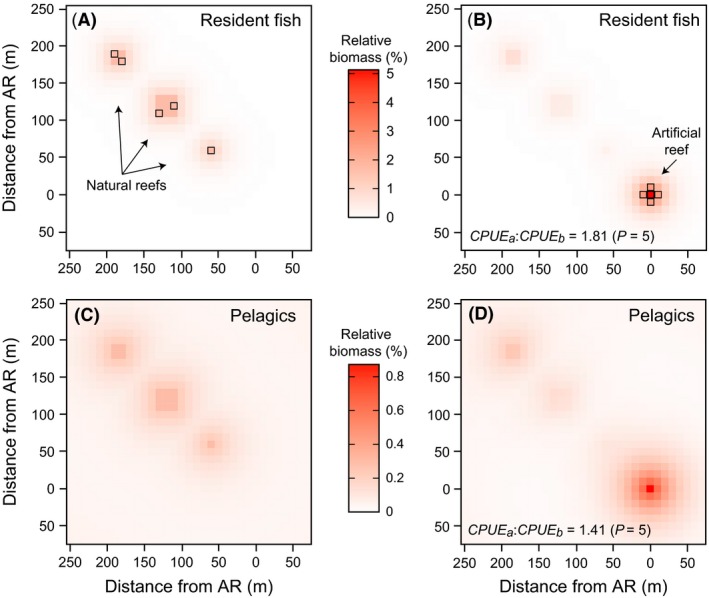
A hypothetical example of fish distribution and attraction in two dimensions, for both reef‐resident fish (A, B) and reef‐associated pelagics (C, D). Fish biomass is distributed across three natural reefs of varying sizes, 1 × 1 cell, 2 × 2 cells, and 3 × 3 cells (A, C); this biomass is attracted to a 1 × 1 cell artificial reef and redistributed around this reef (B, D). The change in CPUE due to this attraction can be observed by calculating the fish biomass in *p* cells, before and after reef deployment. In this example, *p *=* *5 (A, cells outlined), and the fished cells change after fish attraction to follow the maximum fish biomass (B, cells outlined). CPUE increased for both reef residents and pelagics after the artificial reef was added. In this example, the parameter values were *Q *=* *5, *D*_*h*_ = 10, *D*_50_ = 300, and *D*_99_ = 600 (residents); and *Q *=* *5, *Q*_*nr*_ = 1, *D*_*h*_ = 20, *D*_50_ = 200, and *D*_99_ = 400 (pelagics).

Simulations using the above model were performed to identify the scenarios (in terms of reef and fish characteristics) for which the risk of exploitation of fish biomass was highest. The parameters varied in this simulation were as follows: *D*_*h*_, *Q*,* D*_50_, *D*_*r*_, and *p* (Table 1). In addition, the size of the natural reef (*S*_*r*_) was varied at three levels (1 × 1 cell, 2 × 2 cells, and 3 × 3 cells) to account for the increased fish biomass available for attraction on larger reefs, and to account for the increased number of reef cells that can be simultaneously fished on larger reefs. The artificial reef was kept constant at 1 × 1 cell. These six factors were systematically varied to illustrate numerous possible scenarios and fish responses; for example, an artificial reef placed near to a large natural reef of much poorer quality attracts more fish than an artificial reef placed far from a smaller natural reef of equal quality. Simulations were performed for both reef‐resident species (which have a biomass = 0 in the absence of a reef) and reef‐associated pelagic species (which have a biomass > 0 in the absence of a reef). Figure 2 illustrates a similar system to that used in the simulations, in which the depletion of fish biomass due to attraction to an artificial reef can be observed, with nearby reefs having higher depletion than more distant reefs.

The results of simulations are best presented graphically due to some clear nonlinearities. Given that each simulated scenario is a function of six variables, multiple sets of 2‐dimensional plots are required to communicate results. A sensitivity analysis was also performed as an alternative illustration of parameter importance. The levels of each parameter were varied randomly in a Monte Carlo simulation, and the CPUE ratio stored at each iteration (the levels used were those in Table 1, except *D*_50_ which had 5 levels between 100 and 300 m). This was iterated 2000 times, and a linear model fitted to the resulting dataset (Smith et al. 2012) using parameters standardized according to Kleijnen (1997). Due to the nonlinearities mentioned above, results are reported separately for distances with high attraction from the natural reef (*D*_*r*_ = 42 m), some attraction (*D*_*r*_ = 250 m), and little attraction (*D*_*r*_ = 510 m).

## Results

### Attraction of fish biomass

**Figure 3:**
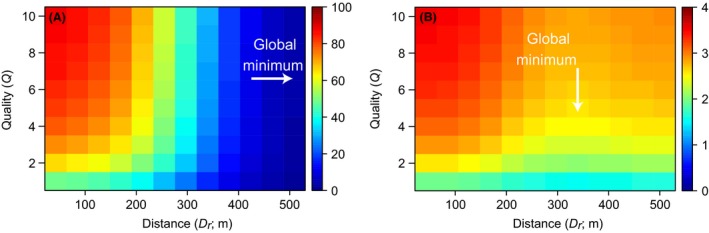
The typical redistribution of the existing biomass for reef fish (A) and reef‐associated pelagics (B) in the entire modeled system (2.1 × 2.1 km), in terms of distance *D*_*r*_ and relative quality *Q*. Color is the percentage of total fish biomass in the system that was attracted to the artificial reef. The percentage is much higher for reef fish as their biomass is restricted to the area around either reef, whereas pelagic fish biomass can exist throughout the entire simulated area (much of which is outisde the influence of the natural or artificial reefs). The global minimum of attracted fish biomass for reef residents occurs when artificial and natural reefs are farthest apart (A), but occurs at a closer distance for reef‐associated pelagics (B) due to an overlap of the “attraction halos” for the natural and artificial reefs. The fixed parameter values in these examples were *D*_50_ = 300, *D*_99_ = 600, *D*_*h*_ = 10, *p *=* *5, and *S*_*r*_ = 2 × 2 (A); and *D*_50_ = 200, *D*_99_ = 400, *D*_*h*_ = 20, *Q*_*nr*_ = 1, *p *=* *5, and *S*_*r*_ = 2 × 2 (B).

As expected, the biomass of reef‐resident fish attracted to an artificial reef declined with increasing distance between reefs and increased with increasing quality of the artificial reef (Fig. 3A). The attracted biomass of reef‐associated pelagic fish also increased with increasing reef quality but, unlike reef residents, attracted biomass was lowest at an intermediate distance between reefs (Fig. 3B). This shows an important difference between the two functional fish groups: attracted biomass will always be lowest for reef residents on very isolated reefs, but lowest for reef‐associated pelagics when the “attraction halo” of the artificial reef overlaps the halo of an existing nearby reef.

### Impact of attraction on CPUE

**Figure 4:**
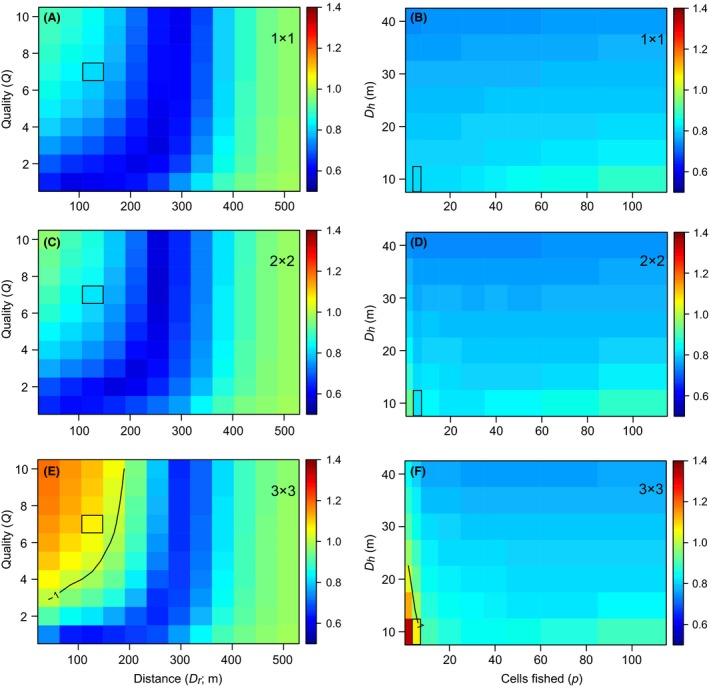
Evaluation of the impact of attraction for reef fish; color is the *CPUE*_*a*_: *CPUE*_*b*_ ratio. A black contour line indicates when *CPUE*_*a*_: *CPUE*_*b*_ equals 1. For most scenarios modeled this ratio <1, meaning that CPUE actually declined after the addition of the artificial reef. The model was designed to evaluate parameters bivariately; thus, a common cell is shared between each pair of figures for a given reef size; this cell is outlined in black. Thus, the change in the CPUE ratio in terms of *D*_*h*_ and *p* for the outlined cell in (A) is shown in (B). The fixed parameter values for A, C, and E were *D*_*h*_ = 10, *D*_50_ = 300, *D*_99_ = 600, and *p *=* *5; and for B, D, and F were *Q *=* *7, *D*_*r*_ = 127, *D*_50_ = 300, and *D*_99_ = 600.

The simulations revealed some nonlinear patterns between *CPUE*_*a*_: *CPUE*_*b*_ and model parameters. It can be seen that the change in CPUE is lowest for reef residents at intermediate distances between reefs, that is, when partial attraction occurs (Fig. 4A,C,E). For natural reefs equal (Fig. 4A) or four times larger (Fig. 4C) in size than the artificial reef, CPUE actually decreased when the artificial reef was added for every scenario of distance and quality. This held true for the scenarios tested for variation in *D*_*h*_ and in number of cells fished *p* (Fig. 4B,D). Only when natural reef size was nine times greater than the area of artificial reef did CPUE increase due to the artificial reef (Fig. 4E), with the largest increase occurring for small distances and high quality. This appeared to be largely restricted to scenarios with small *D*_*h*_ and small *p* (Fig. 4F). The CPUE ratio approached 1 (i.e., unchanged CPUE) as both distance *D*_*r*_ and fishing effort *p* became large.

**Figure 5:**
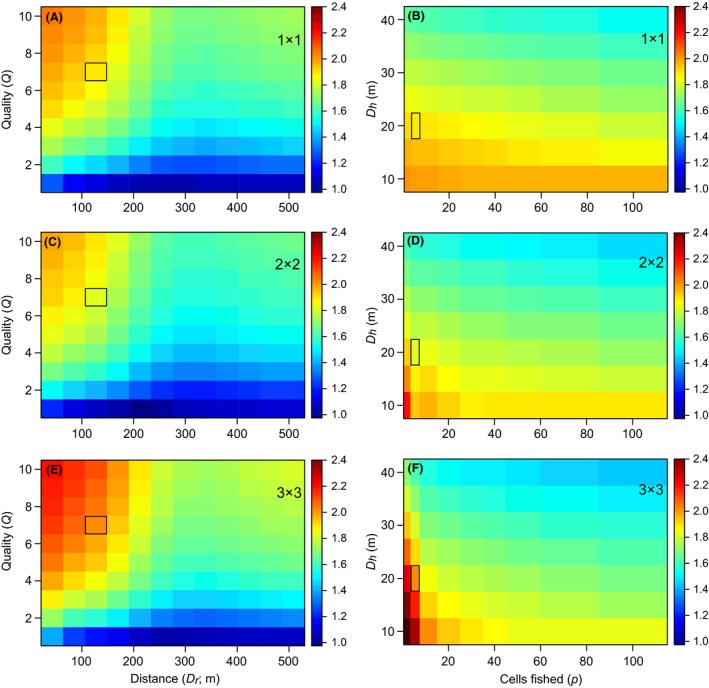
Evaluation of effects of “attraction” for reef‐associated pelagic fish; color is the *CPUE*_*a*_: *CPUE*_*b*_ ratio. The bottom left cell in F) exceeded the chosen color scale and has value of 3.03. The fixed parameter values for A, C, and E were *D*_*h*_ = 20, *D*_50_ = 200, *D*_99_ = 400, and *p *=* *5; and for B, D, and F were *Q *=* *7, *D*_*r*_ = 127, *D*_50_ = 200, and *D*_99_ = 400. See the Fig. 4 caption for more information.

Reef‐associated pelagic fish showed a similar response. CPUE changed most for larger natural reefs and when the artificial reef was closest and of greatest relative quality (Fig. 5A,C,E), plus when *D*_*h*_ was small and fishing effort was low (Fig. 5B,D,F). Unlike the reef‐resident group, however, the CPUE for pelagics was almost always >1. Like reef residents, the CPUE ratio for pelagics approached 1 as *p* became large, but, unlike reef residents, the CPUE ratio did not approach 1 for large *D*_*r*_, reflecting differences in the attraction of fish biomass (Fig. 3). The apparent decline in CPUE ratios for the 2 × 2 reef (Fig. 5C) was due to inefficient CPUE occurring when *p* is the same as the number of cells containing reef. Parameter‐specific findings are summarized in Table 2.

**Table 2:** Parameter‐specific simulation and sensitivity analysis parameter results

Parameter	Result
*D*_*h*_	Fish with tighter associations with reef (small *D*_*h*_) at higher risk of exploitation; this is because they are easier locate; always inversely related to the CPUE ratio
*Q*	Increased relative quality of artificial reef increases risk of exploitation – this was by design; a more important driver for transient fish than reef fish due to the increased area of attraction for transient fish
*D*_50_	As *D*_50_ increases, the area from which an artificial reef attracts fish increases, and thus, reefs must be more isolated to reduce attraction (same goes for *D*_99_); simulations reveal that increasing *D*_50_ linearly increases the distance at which the minimum change in CPUE ratio occurs (data not shown); can inversely influence CPUE ratio at intermediate distances for reef residents as it drives partial attraction (and a decline in the CPUE ratio)
*D*_*r*_	*For reef fish*: an increasing distance between artificial and natural reefs means reduced attraction, to an attracted biomass of zero at large distances; minimum change in CPUE ratio occurs with partial attraction, but no change (ratio = 1) will always occur at large distances *For pelagics*: the minimum biomass attraction and the minimum change in CPUE occurs at intermediate distances where the “halos” of attracted biomass for both reefs overlap, but partial attraction less important for determining patterns of attraction than for reef fish
*S*_*r*_	Increasing the relative size of natural reef increases risk of exploitation, because more biomass is available for concentration by the artificial reef; to ensure an equal biomass density after attraction on natural and artificial reefs *D*_*r*_ must increase as *S*_*r*_ increases; less influential than *Q*,* D*_*h,*_ or *D*_50_
*p*	An increase in CPUE most likely for small fishing effort *p*; as fishing effort increases, the CPUE ratio approaches 1; consider that the CPUE ratio does not represent change in absolute biomass harvested, which instead peaks at intermediate values of *p*

**Figure 6:**
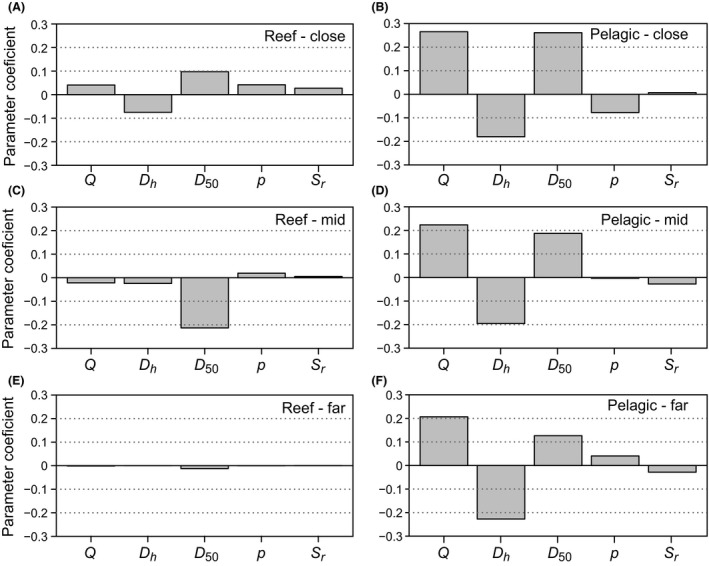
Results of sensitivity analyses examining relative parameter importance on the CPUE ratio, for reef residents (A, C, E) and reef‐associated pelagics (B, D, F). Due to the nonlinearities in the response of the CPUE ratio across distance, these have been split into parameter effects at a close distance with high fish attraction (*D*_*r*_ = 42 m), a mid‐distance with partial attraction (*D*_*r*_ = 255 m), and a far distance with low attraction (*D*_*r*_ = 509 m). The parameter coefficients are those of the linear model on standardized data, but the value is arbitrary and the relative bar height is used to determine relative parameter importance.

The sensitivity analysis showed that all parameters become less influential on the CPUE ratio as distance from NR (*D*_*r*_) increases for reef residents (Fig. 6A,C,E), due to the declining attracted fish biomass. Mobility (*D*_50_) does become the most influential at intermediate distances, due to its importance for influencing partial attraction (and hence inversely the CPUE ratio) at these distances. For reef‐associated pelagics (Fig. 6B,D,F), we do not see a decline in parameter importance with distance (unlike reef residents), because pelagics are also attracted to the AR from the surrounding pelagic environment. Reef quality (*Q*), fidelity (*D*_*h*_), and mobility (*D*_50_) are the most influential parameters on the CPUE ratio, but fidelity becomes more influential as distance increases, because attraction from the NR declines and fidelity (inversely) drives the CPUE ratio by influencing how easily fish are located. Generally, it is the properties of the species that are generally most important (mobility and fidelity) for driving change in CPUE, followed by the attractiveness of the AR. Surprisingly, neither fishing intensity (*p*) nor natural reef size (*S*_*r*_) was as influential over the spatial and parameter scales tested here.

To test the general effect of a threshold CPUE (i.e., maximum CPUE is achieved at a threshold fish density), rather than a linear relationship between CPUE and fish density, the model was run with maximum CPUE occurring at 70% of the maximum fish density observed before the artificial reef was deployed. Doing so increased the area in which maximum CPUE occurred, which meant that the CPUE ratio in many scenarios was unchanged (=1) or approached this value (Figs S1–S2), especially for low fishing effort (*p*). This means that a threshold or asymptotic relationship between CPUE and fish density reduces the effect of both partial attraction and concentration, by maintaining a more constant catch rate across a broader range of scenarios.

## Discussion

Evaluation of our attraction model reveals some important generalities. In terms of exploitation of fish biomass, the typically maligned process of attraction is not always bad; in fact, attraction can sometimes disperse existing fish biomass instead of concentrating it, thus making it harder to exploit. There remain numerous scenarios under which attraction does create opportunities to exploit fish biomass, and, in general, maximum risk occurs given high‐quality artificial reefs very close to existing reefs, for species with low *D*_*h*_, and when fishing is restricted to cells with highest fish biomass (i.e., low‐moderate *p*). Although reef residents and reef‐associated pelagics showed different patterns of attraction, they shared this pattern of maximum risk. The greatest dispersion of fish biomass, and thus the largest reduction (or smallest increase) in CPUE, occurred when there was partial attraction, and this was true for both functional groups. Our results show that attraction is a complex process that is not always harmful, that the role of attraction in influencing the success of artificial reefs is a function of reef, species, and harvest characteristics, and that the fidelity and mobility of the attracted fish species may be the key drivers of the outcomes of harvesting attracted fish biomass.

### Identifying risky scenarios

Table 2 summarizes the scenarios in which risk of exploitation of fish biomass due to attraction is highest. As expected, attraction is greatest when an artificial reef is of high quality and deployed close to a large area of existing reef. The species most likely to be exploited are those that are closely associated with a reef (low *D*_*h*_) because they are easier to locate. Reef‐associated pelagics are attracted under all scenarios, and their CPUE increases in almost all scenarios. Their often patchy abundance may reduce this risk in the long term, however, and only a part of their biomass is associated with a reef at any one time (as opposed to reef residents). Most interestingly, the least risk for either functional fish group occurred when there was partial attraction. The ideal level of partial attraction is when the dispersion of fish biomass is most likely (as opposed to the concentration of fish biomass) and generally coincided with an equivalence of fish biomass on the artificial and natural reefs. The distance between artificial reefs and existing reefs is one factor over which fisheries managers have some control, and selecting the distance that ideal level of partial attraction will reduce the risk of attraction in an artificial reef deployment. If the system is too data poor to estimate the distance at which this level of attraction occurs, then a greatly isolated artificial reef may be the best choice as this reduces attraction for obligate reef residents (Carr and Hixon 1997). If highly mobile reef‐associated species are of greater concern, then a distance between reefs that encourages partial attraction is the best option, even in data‐poor systems. It is when these highly mobile species must make a choice between the natural reef and the artificial reef that concentration of existing fish biomass is minimized.

### Examining bohnsack's predictions

Factors that might increase fish attraction (or the harm from fish attraction) were proposed early on in artificial reef research by Bohnsack (1989), and we were able to compare some of these predictions with our model's predictions. Bohnsack predicted that attraction would be a dominant process when reef availability was high. Our findings agree with this, such that attraction is more likely given reef in close proximity to an artificial reef, and also that the greater the reef area exposed to attraction, the more harmful attraction can be. Bohnsack predicted that high fishing intensity increases the risk of attraction. Our study shows that this is a complicated factor and that a low fishing intensity will cause the largest relative increase in CPUE, but the actual harvest increases most at moderate fishing intensity. In fact, at highest fishing intensity, when poor quality areas are also being fished, a redistributed fish biomass makes little difference as most fish are caught wherever they are. Bohnsack also predicted that more mobile mid‐water species are at more risk of attraction than territorial or demersal species. Our findings somewhat agree, such that our “pelagics” are generally at more risk and that an increasing willingness to move between reefs (large *D*_50_) would also increase risk. Our findings show, however, that a tighter association with a reef (smaller *D*_*h*_) puts you at greater risk of exploitation, which may be at odds with Bohnsack's prediction. And finally, Bohnsack predicted that species loosely associated with reefs are at more risk from attraction than reef obligates. Our findings generally agree, such that reef‐associated pelagics are attracted in most scenarios, even when no natural reefs are nearby. But again it is important to distinguish between the roles of mobility (*D*_50_) and fidelity (*D*_*h*_), and we found that reef residents are probably easier to exploit given that all their biomass is easily located (not just their attracted biomass). Our recommendation is to improve the scenarios given by Bohnsack by specifying the mobility and habitat traits of species in terms of a willingness to move to a new reef (mobility, *D*_50_) and the scale of the association with a reef (fidelity, *D*_*h*_), as these have clear, numeric identities, and are generally the most influential parameters across a range of scenarios.

### Model and conceptual considerations

This study identified scenarios that have the highest risk of harmful attraction. However, some of these scenarios may also be those that would maximize production (e.g., an artificial reef in a tight mosaic of existing natural reef). This means that one might inadvertently reduce the potential for fish production by deliberately reducing the risk of possible attraction. Unfortunately, this will remain the case until the processes that drive fish production are better identified and modeled. It is also the case that not in some management scenarios fish attraction may be not considered harmful, such as artificial reefs deployed in protected areas (Brochier et al. 2015). These cases highlight that the spatial distribution of fishing effort can be as important as fish biomass for understanding the risks associated with fish attraction. Generally, however, we consider any artificial reef exposed to fishing will have some risk of increasing fisheries harvest, regardless of a reef's specific management objectives.

This lack of understanding about fish production, and the difficulty of distinguishing production and biomass redistribution, reduces our ability to identify the true risk of fish attraction. It is likely that the driving factors of the attraction modeled in this study go beyond “instinctive orientation” (Pickering and Whitmarsh 1997), “thigmotaxis” (Brickhill et al. 2005), and “behavioral preferences” (Bohnsack 1989), to include factors that provide very real benefits to survival or food availability, even when colonization happens quickly (Brandt and Jackson 2013). This means that there will be changes to survival and/or growth that must be considered production, even though it was only existing fish biomass that responded to the new reef. It is imperative that we continue to strive to identify fish production, including benefits to existing fish biomass, if we are truly understand the effects of deploying artificial reefs.

The sensitivity analysis showed that our model is greatly influenced by the spatial parameters *D*_*h*_ and *D*_50_, especially for transient species. The *D*_50_ distances evaluated in this study (100–300 m) were kept small for model tractability and probably underestimate the ability of many species to move to new habitat, although there is some evidence that reefs <600 m to 1 km apart show reduced connectivity (Bohnsack and Sutherland 1985; Chang 1985; Folpp et al. 2013). Without accurate knowledge of these parameter values, our model cannot be used predictively to determine how far an artificial reef should be deployed from natural reefs. However, the generalities identified in the model are largely independent of species parameter values and combining observations of nearby natural reefs with the recommended target of equivalent biomasses on artificial and natural reefs after attraction gives our findings practical value.

Our model made some assumptions of note. It was assumed that CPUE was linearly related to fish biomass density to model the worst case scenario, but simulating a threshold relationship showed that the risk from attraction will decrease for many scenarios, as catch rates will be more similar before and after an artificial reef is deployed. Accounting for density dependence in the fish density–CPUE relationship is obviously essential for modeling specific systems. It is likely that density dependence would also influence our “reef quality”. An artificial reef may decline in quality for some species as biomass on that reef increases. If this model was to be used for larger areas of natural reef (larger than the 9:1 area modeled here), attracted biomass may be overestimated. However, we believe that even without this density‐dependent attraction, our patterns of risk in terms of distance and fishing effort are robust.

It is assumed in our model that fishing effort is equivalent before and after reef deployment. If fishing effort increases due to a reef's deployment, and some have argued that artificial reefs may increase fishing effort on hard substrates in general (Grossman et al. 1997), then risk from attraction increases beyond model predictions. It is also possible that much fishing effort will move from outside to inside the modeled system after reef deployment. The decline in catch outside the system due to this redistributed effort must be estimated whether the true risk of attraction is to be modeled; that is, the risk of attraction is not represented by the total harvest from an artificial reef, but by the change in harvest due to redistributed (and new) fishing effort. It is thus clear that the redistribution of fishing effort is as important as the redistribution of fish biomass for estimating the risk, effects, and impacts of artificial reefs.

## Conclusion

There is unlikely to ever be a one‐size‐fits‐all model of the value of artificial reefs to fish production, given the possible variation in reef design, location, target fish species, and site‐specific food and larval supply. However, we believe that attraction is not as harmful a process as is generally reported, because it does not always increase maximum fish density. This lessens the burden to create models of fish production before a reef can be responsibly deployed. What is more, if bag limits as a management strategy is used and enforced, and fishers are generally reaching their bag limits, then attraction that does increase maximum fish density may simply result in reduced trip times and increased angler satisfaction. Plus, if an increase in fisheries harvest in a system is sustainable, an increase in fish density due to attraction may not be harmful at all. It may be that the most effective method to reduce exploitation is to encourage sustainable harvest through monitoring and management action (Powers et al. 2003; Simon et al. 2011), particularly while fish production remains difficult to estimate. A precautionary measure may be to close artificial reefs to fishing for an initial period (Brickhill et al. 2005). Given that fish attraction and production are ends of the same spectrum (Bohnsack 1989), a goal may be to create spatially explicit and multispecies individual based models that can integrate these two processes, but until then we must rely on species‐specific studies and single‐process models, and the most process‐driven analysis of fish attraction will be one that distinguishes between the dispersion and concentration of existing fish biomass. To continue to deploy artificial reefs, we must accept that engineering reef habitat will always have consequences for marine life and almost certainly cause a redistribution of some fish biomass, but we can now acknowledge that redistribution is not necessarily harmful.

## Conflict of Interest

None declared.

## Supporting information


**Figure S1** Evaluation of the impact of attraction for reef fish, with maximum CPUE occuring at 70% of the maximum fish density before artficial reef deployment.
**Figure S2** Evaluation of the impact of attraction for reef‐associated pelagic fish, with maximum CPUE occuring at 70% of the maximum fish density before artficial reef deployment.
