# Supplementary material for: Fish attraction to artificial reefs not always harmful: a simulation study
Source: Ecol Evol. 2015 Sep 30;5(20):4590–602. doi: 10.1002/ece3.1730 (PMC4670052; doi:10.1002/ece3.1730)
Supplement: Supplementary file 1 — Figure S1 Evaluation of the impact of attraction for reef fish, with maximum CPUE occuring at 70% of the maximum fish density before artficial reef deployment. Figure S2 Evaluation of the impact of attraction for reef‐associated pelagic fish, with maximum CPUE occuring at 70% of the maximum fish density before artficial reef deployment. [file ECE3-5-4590-s001.docx]

**Supporting Information:**


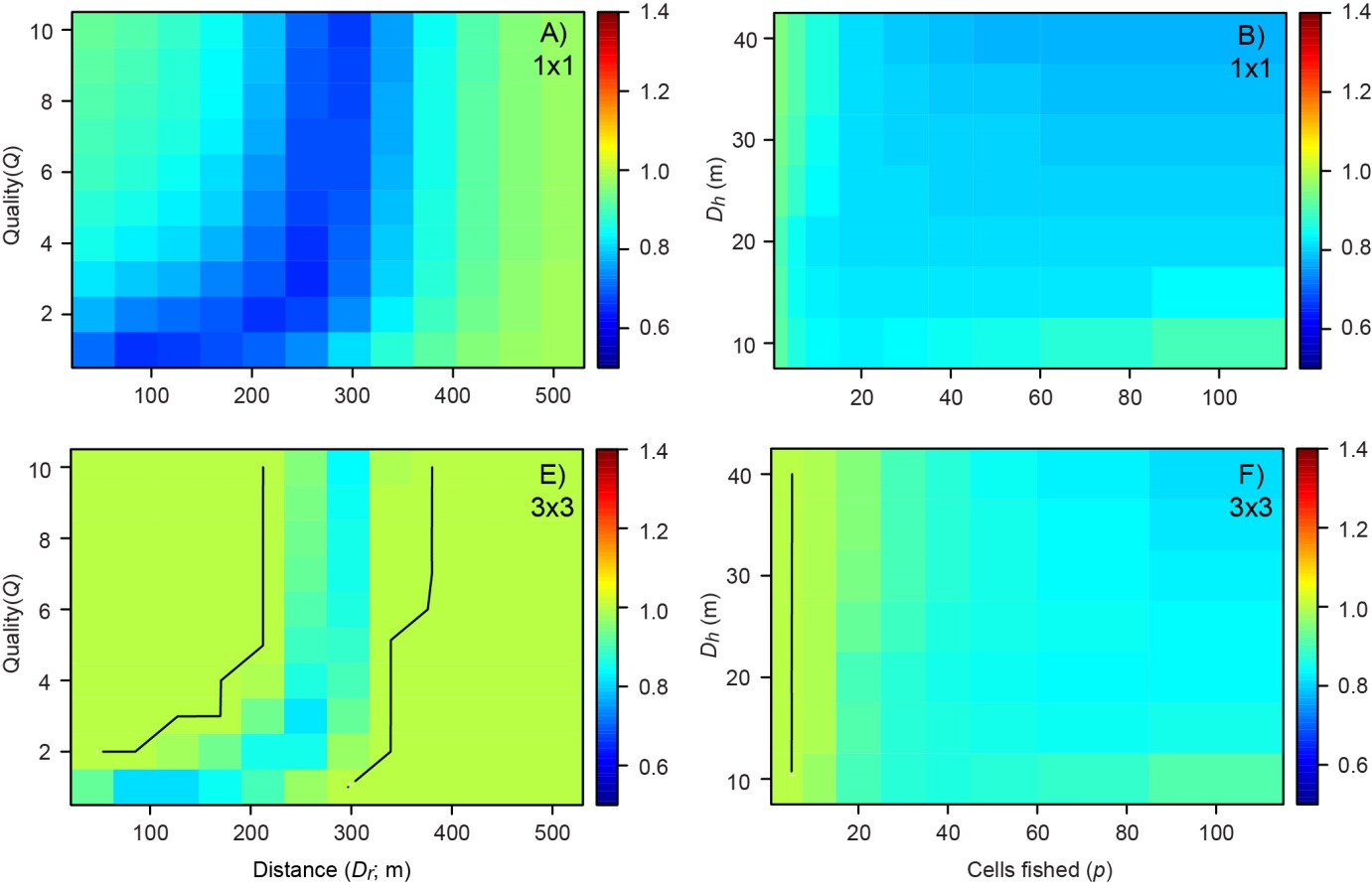


**Figure S1:** Evaluation of the impact of attraction for reef fish, with maximum CPUE occuring at 70% of the maximum fish density before artficial reef deployment. Colour is the *CPUE_a_*:*CPUE_b_* ratio, and the contour lines indicate where this ratio = 1. This was done for *S_r_* = 1x1 cell and *S_r_* = 3x3 cells. For direct comparison with Fig. 4 in the main article. The fixed parameter values for A, E were: *D_h_* = 10, *D*_50_ = 300, *D*_99_ = 600, *p* = 5; and for B, F were: *Q* = 7, *D_r_* = 127, *D*_50_ = 300, *D*_99_ = 600.


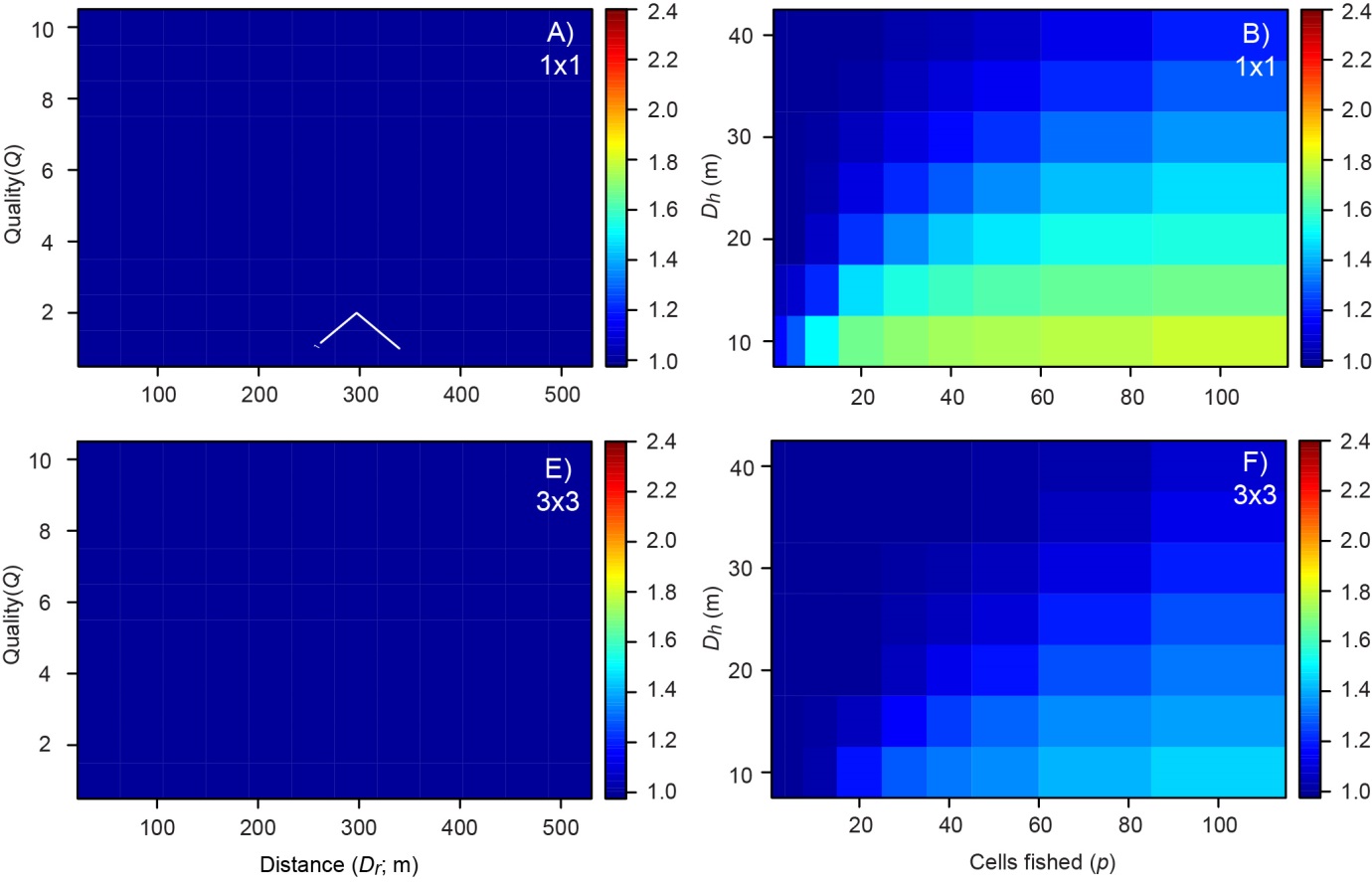


**Figure S2:** Evaluation of the impact of attraction for reef-associated pelagic fish, with maximum CPUE occuring at 70% of the maximum fish density before artficial reef deployment. Colour is the *CPUE_a_*:*CPUE_b_* ratio, and the contour line indicates where this ratio = 1. This was done for *S_r_* = 1x1 cell and *S_r_* = 3x3 cells. For direct comparison with Fig. 5 in the main article. The fixed parameter values for A, E were: *D_h_* = 20, *D*_50_ = 200, *D*_99_ = 400, *p* = 5; and for B, F were: *Q* = 7, *D_r_* = 127, *D*_50_ = 200, *D*_99_ = 400.
